# Structural, Thermal, and Mechanical Characterization of a Thermally Conductive Polymer Composite for Heat Exchanger Applications

**DOI:** 10.3390/polym13121970

**Published:** 2021-06-15

**Authors:** Jamieson Brechtl, Yuzhan Li, Kai Li, Logan Kearney, Kashif Nawaz, Alexis Flores-Betancourt, Michael Thompson, Orlando Rios, Ayyoub M. Momen

**Affiliations:** 1 Multifunctional Equipment Research Group, Oak Ridge National Laboratory, Oak Ridge, TN 37831, USA; yuzhanli@ustb.edu.cn (Y.L.); lik1@ornl.gov (K.L.); ayyoubmomen@ultratechsol.com (A.M.M.); 2 School of Materials Science and Engineering, University of Science and Technology Beijing, Beijing 100083, China; 3 Chemical Sciences Division, Oak Ridge National Laboratory, Oak Ridge, TN 37831, USA; kearneylt@ornl.gov; 4 Materials Science and Technology Division, Oak Ridge National Laboratory, Oak Ridge, TN 37831, USA; floresbetana@ornl.gov; 5 Department of Materials Science and Engineering, The University of Tennessee, Knoxville, TN 37996, USA; mthom133@vols.utk.edu (M.T.); orios1@utk.edu (O.R.)

**Keywords:** polymer composites, microstructure analysis, thermal analysis, creep modeling, mechanical properties

## Abstract

Polymer composites are being considered for numerous thermal applications because of their inherent benefits, such as light weight, corrosion resistance, and reduced cost. In this work, the microstructural, thermal, and mechanical properties of a 3D printed polymer composite with high thermal conductivity are examined using multiple characterization techniques. Infrared spectroscopy and X-ray diffraction reveal that the composite contains a polyphenylene sulfide matrix with graphitic fillers, which is responsible for the high thermal conductivity. Furthermore, differential scanning calorimetry determines that the glass transition and melting point of the composite are 87.6 °C and 285.6 °C, respectively. Thermogravimetric analysis reveals that the composite is thermally stable up to ~400 °C. Creep tests are performed at different isotherms to evaluate the long-term performance of the composite. The creep result indicates that the composite can maintain mechanical integrity when used below its glass transition temperature. Nanoindentation tests reveal that modulus and hardness of the composite is not significantly influenced by heating or creep conditions. These findings indicate that the composite is potentially suitable for heat exchanger applications.

## 1. Introduction

Polymer composites are versatile materials and have been used in many applications, including construction [1,2,3], oil field [4], energy [5,6], transportation [7], and automation etc. [8,9,10,11,12]. With interest in cost-effective and durable energy conversion systems growing, there is an immense focus on the deployment of polymer-based solutions. Applications such as waste heat recovery, power generation, thermal desalination, and air conditioning are some obvious examples for which polymer and polymer composite materials are being considered and, in several cases, successfully deployed to provide sustainable performance. Waste heat is a form of energy that is a byproduct of almost all mechanical and thermal processes. This type of energy is produced during industrial processes. Globally, approximately 247 PJ of energy is discharged as waste heat in exhaust gas and effluents [13]. In the United States, the waste heat resulting from industrial emissions accounted for ~4.3% of the total energy use by industry as of 2015 [14,15]. More than 80% of this energy was in the low-temperature regime, which corresponds to temperatures ranging from 25 to 150 °C [15]. Although metallic low-temperature heat recovery systems are available, they suffer from issues such as high material costs and long payback periods [14]. 

An alternative to a metallic thermal recovery system is a polymeric heat exchanger. Compared with other materials (metal alloys and ceramics) deployed in conventional technologies, polymer-based solutions provide novel characteristics such as higher specific capacity (kW/kg), opportunities to modulate designs, and effective resistance to material degradation due to corrosion. A polymer is typically constructed through a chemical reaction between monomers, resulting in the formation of a long chain composed of many repeated organic subunits [16]. The wide variety of monomers and reaction types enables great design flexibility for the control of structure and properties. Furthermore, polymeric materials have desirable properties such as low-temperature processing, shaping, and joining; resistance to fouling; and corrosion resistance [17]. In terms of corrosion resistance, polymeric materials such as polyvinylidene fluoride (PVDF), Teflon, and polyphenylene sulfide can accommodate highly corrosive environments during exposure to appreciable mechanical stresses because of their high molecular weight and crystalline nature [9,10,11,12,16,18]. For example, Teflon is resistant to practically all corrosives except fluorine and molten alkali metals and can withstand temperatures and pressures of up to 204 °C and 862 kPa, respectively [18]. Moreover, heat exchangers composed of polymers have significantly lower manufacturing costs. For example, Zaheed and Jachuck [18] found that a heat exchanger composed of PVDF would cost ~40% as much as one composed of a Ni-Cr-Mo alloy, considering the differences in material cost and density [19].

Despite these positive aspects, there are some major disadvantages associated with the use of polymers in heat exchanger applications. For example, the lower thermal conductivity of polymers puts them at a disadvantage compared with steel for use in environments typical of a heat exchanger [20]. In addition, the long-term performance of polymer heat exchangers is an important aspect to consider for practical applications. The inherent viscoelastic properties of polymers make them fundamentally different from other materials. The mechanical properties of polymers, such as strength and modulus, change with time because they exhibit characteristics of both an elastic solid and a viscous fluid [21].

In the current study, we focus on characterizing a 3D printed polymer composite with high thermal conductivity and evaluating its potential use for heat exchanger applications. The microstructure of the material was characterized using infrared spectroscopy and X-ray diffraction. We also evaluate the long-term performance of the material using the time–temperature superposition principle based on short-term creep experiments at different isotherms. The objective of this study is twofold: to provide the critical attributes of the material for long-term performance in a typical heat exchanger application and to develop a framework for the compatibility for a range of potential materials that can be considered for such an application. 

## 2. Materials and Methods

### 2.1. Materials and Processing

**Figure 1:**
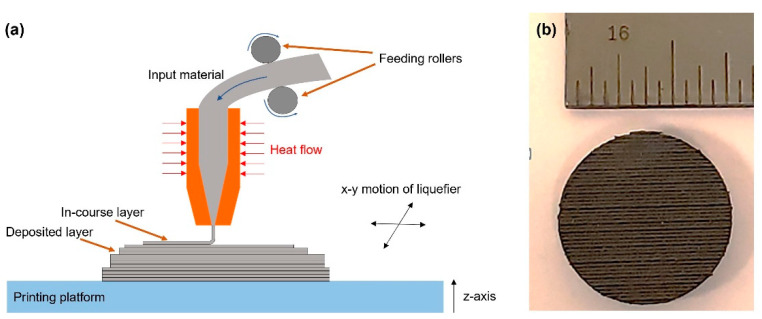
(**a**) Schematic representation of a (FFF) process (adapted from Ref. [22]) and (**b**) 3D printed puck used to fabricate test samples (ruler units are in inches).

The filament used for 3D printing was acquired from a commercial vendor. The material was commercialized as a high thermal conductivity composite (thermal conductivity ~4 W/m × K). Samples (50 × 50 × 3.18 mm^3^) were additively manufactured using fused filament fabrication (FFF) on a MakerGear M2 3D printer. Figure 1a,b presents a schematic representation of a typical FFF process and the printed puck that was used to fabricate the test samples. The nozzle temperature and diameter, filament diameter, and the layer height were selected to be 245 °C, 0.75 mm, 1.75 mm, and 0.45 mm, respectively, with 25% overlap. Furthermore, the heated bed temperature was 75 °C and the print speed was around 30 mm per second. These parameters are critical because they can impact the characteristics of the resulting product. They were adopted to ensure that specific geometrical features could be achieved and could be considered representative parameters for a typical fused filament fabrication process. 

### 2.2. Structural Analysis

The structure of the composite was characterized using X-ray diffraction (XRD) and Fourier-transform infrared spectroscopy (FTIR). XRD revealed crystallinity of the polymer matrix and graphitic filler, whereas FTIR provided information on the chemical composition of the composite. The XRD experiment was performed using a PANalytical X’Pert Pro diffractometer. The X-rays were generated at 45 kV/40 mA, and the wavelength of the X-ray beam was l = 1.541 Å (Cu Ka radiation). The diffraction spectrum was collected over a 2θ range from 10 to 70° at a scan rate of 0.15°/min. The FTIR characterization was conducted using a NICOLET iS50 system in the attenuated total reflectance (ATR) mode. Diamond was used as the ATR crystal. The FTIR spectrum was collected at 4 cm^−1^ resolution using 64 scans from 4000 to 400 cm^−1^ and corrected against ambient air as the background. Small-angle and wide-angle X-ray scattering (SAXS/WAXS) measurements were carried out on a Xenocs Xeuss 3.0 instrument equipped with D2+ MetalJet X-ray source (Ga Kα, λ = 1.341 Å) to investigate possible preferred orientation and aggregation of the fillers. The sample was aligned perpendicular to the direction of the X-ray beam (transmission mode) and the scattered beam was recorded on a Dectris Eiger 2R 4M hybrid photon counting detector with a pixel dimension of 75 × 75 μm^2^. The collected 2-dimensional (2D) SAXS/WAXS images were circularly averaged and expressed as intensity versus q, where q = (4π sin θ)/λ after subtraction of background scattering. The SAXS and WAXS data were collected at the sample-to-detector distance of 1800 and 55 mm, respectively. The surface morphology of the material was characterized using a Hitachi 4700-S scanning electron microscope (SEM). The polymer was carbon coated to keep it from charging, and a 10 keV accelerating voltage and 20 A of current were used. Three-minute scans were used to generate the composition maps. 

### 2.3. Thermal Analysis

The thermal properties of the composite were characterized using thermogravimetric analysis (TGA), differential scanning calorimetry (DSC), dynamic mechanical analysis (DMA), and thermomechanical analysis (TMA). The TGA experiment was performed using a TA Instruments TGA Q50 model. The sample was heated from room temperature to 800 °C at 10 °C/min in a nitrogen atmosphere. The DSC experiment was performed using a TA Instruments DSC 2500 model. A heat-cool-heat cycle was used with a ramp rate of 20 °C/min under a nitrogen atmosphere. The DMA experiment was performed using a TA Instruments Q800 model. In the experiment, a rectangular piece (25 × 12.72 × 3.18 mm^3^) was heated from room temperature to 180 °C at 3 °C/min, under an oscillation frequency of 1 Hz and at an amplitude of 25 mm in dual-cantilever mode. The TMA experiment was performed using a model Q400 thermomechanical analyzer (TA Instruments, Inc., New Castle, Delaware) in expansion mode with a heat-cool-heat cycle at a rate of 3 °C/min. The second heating scan was recorded to calculate the value of coefficient of thermal expansion (CTE).

The creep behavior of the polymer composite was investigated using DMA and the long-term performance was predicted using the time–temperature superposition (TTSP) principle. Creep tests were carried out using the TA Instruments Q800 DMA in dual-cantilever mode. Creep tests were performed using a multi-temperature creep and creep recovery test on a rectangular sample (30 × 3.3 × 1.42 mm^3^). The creep and creep recovery tests were performed at isotherms from 40 to 110 °C in intervals of 10 °C. A constant stress of 0.8 MPa was applied for 10 min, followed by a 20 min recovery period at zero stress. The TTSP principle is commonly used to study the time-dependent mechanical properties of polymers [23,24]. According to the TTSP principle, a creep experiment conducted at an elevated temperature is equivalent to one performed for an extended period of time. Therefore, short-term creep test data collected at different temperature isotherms can be used to construct a master curve that provides a prediction of the long-term performance of a polymeric material [25,26,27]. It is worth mentioning that TTSP exhibits limitations when multi-phase systems are studied, especially in inhomogeneous systems. However, TTSP can be applied to multi-component systems that are homogeneous and isotropic. 

The creep compliance data were fit according to the Burgers’ four-element model [28]. This type of model consists of a series of combinations of the Maxwell and Kelvin–Voigt models [29]. The Maxwell model can be represented by an elastic spring and a viscous damper connected in series, and the Kelvin–Voigt model consists of an elastic spring and a viscous damper in parallel. The general equation for the model is written as [30]:

(1) $$J\left(t\right)=J_{0}+J_{1}\left[1-e^{-{\left(\frac{t}{\tau }\right)}^{\alpha }}\right]+J_{2}t$$

where *t* is the time elapsed after loading, *J*_0_ is the instantaneous creep compliance for small time scales (~1 s^−1^), *J*_1_ is the compliance modulus coefficient associated with the elastic modulus for the Kelvin-Voigt spring and dashpot, *J*_2_ is the compliance coefficient related to the permanent deformation of the sample, *α* is the Kohlrausch coefficient [31] (between 0 and 1), and *τ* is the retardation time. In Equation (1), *J*_0_, *J*_1_, and *J*_2_ represent the elastic, delayed elastic, and viscous behavior of a polymer, respectively, thereby providing a full picture of the viscoelastic response of the material during the creep test. The retardation time indicates the intrinsic viscosity of a polymer, as the transient strain in a creep test occurs as a result of the viscosity of the assembly of molecular chains. Note that permanent deformation of the sample is associated with slipping of the polymer chains relative to one another [30].

### 2.4. Nanoindentation Testing

**Figure 2:**
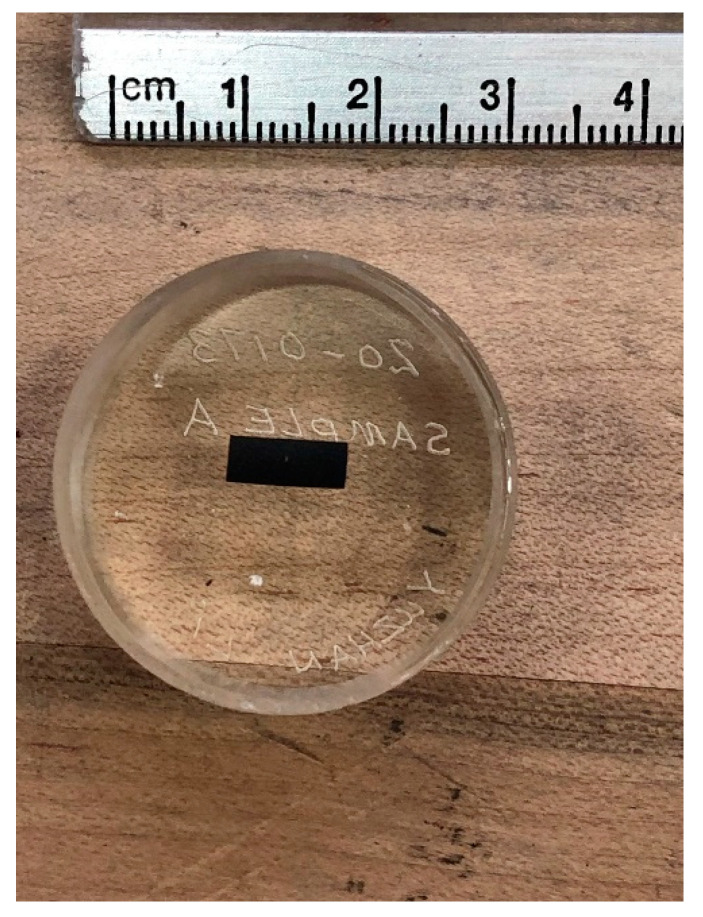
The as-received polymer nanoindentation sample that was cold mounted in epoxy and polished to a mirror finish.

Nanoindentation tests were performed to determine the hardness and Young’s moduli of the samples due to their limited size. Samples were cold mounted in epoxy and polished to a mirror finish (see Figure 2). Indentations were conducted on as-received, heated-without-creep, and heated-with-creep specimens via a Hysitron TriboIndenter TI-900. The tests were performed using a standard load transducer with a Berkovich diamond tip that was calibrated using a standard fused silica sample. A grid of 15 × 15 indents with 50 μm spacing was made on each sample under displacement control mode with a maximum depth of ~1000 nm. During the tests, the tip penetrated the sample until it reached the maximum depth, which was then held for 2 s and subsequently unloaded. The loading and unloading portions of the experiments were ~5 s each. 

## 3. Results and Discussion

### 3.1. Structure Analysis

**Figure 3:**
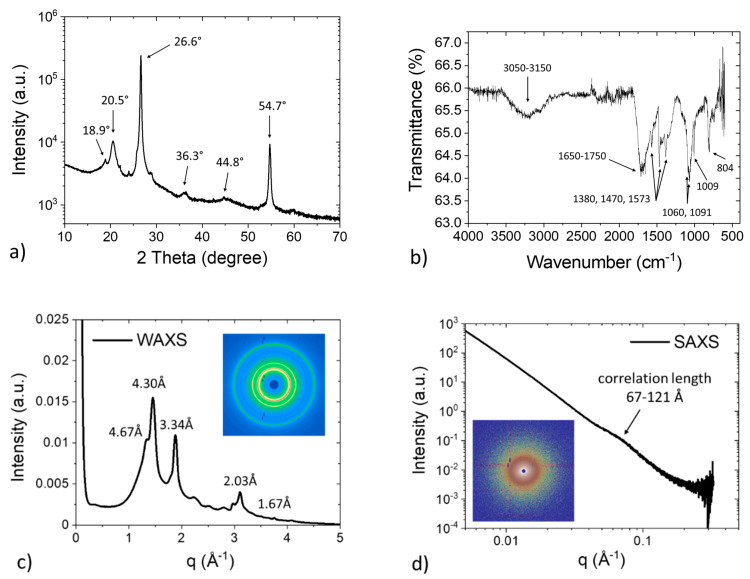
Structural analysis of the polymer composite. (**a**) XRD spectrum indicating a possible composite structure of PPS and graphite; (**b**) FTIR spectrum showing the presence of an aromatic structure with a carbon–sulfur bond; (**c**) 2D WAXS pattern and integrated 1D profile confirming an isotropic nature of the composite; and (**d**) 2D SAXS pattern and integrated 1D profile showing the presence of an assembled structure with correlation lengths between 67 and 121 Å.

The XRD spectrum of the polymer composite is displayed in Figure 3a; the diffraction peaks at scattering angles of 18.9°, 20.5°, and 36.3° suggest that the polymer composite is composed of a polyphenylene sulfide (PPS) matrix [32,33,34]. Furthermore, the diffraction peaks at 26.6° and 54.7° indicate the presence of a graphitic structure that is possibly composed of carbon black, carbon fiber, or carbon nanotubes. To confirm the chemical composition of the polymer matrix, FTIR was utilized and the spectrum, displayed in Figure 3b, shows that stretching and bending vibrations of the aromatic structures, as well as carbon–sulfur stretching vibration, occurred [35]. These results suggest that the sample consisted of a PPS-based polymer matrix. The detailed peak assignments of the XRD and FTIR spectra are summarized in Table 1, confirming the presence of the PPS polymer matrix. Figure 3c shows the 2D WAXS pattern of the polymer composite. The scattering peaks correlate well with the diffraction peaks observed in the XRD spectrum. In addition, the uniform intensity distribution of the scattering rings confirms that the material is macroscopically isotropic. Figure 3d shows the 2D SAXS pattern and the integrated 1D scattering profile of the polymer composite. The shoulder observed in the 1D scattering profile indicates the presence of an assembled nanostructure with correlation lengths between 67 and 121 Å, possibly due to the aggregated graphitic fillers.

### 3.2. Thermal Analysis

**Figure 4:**
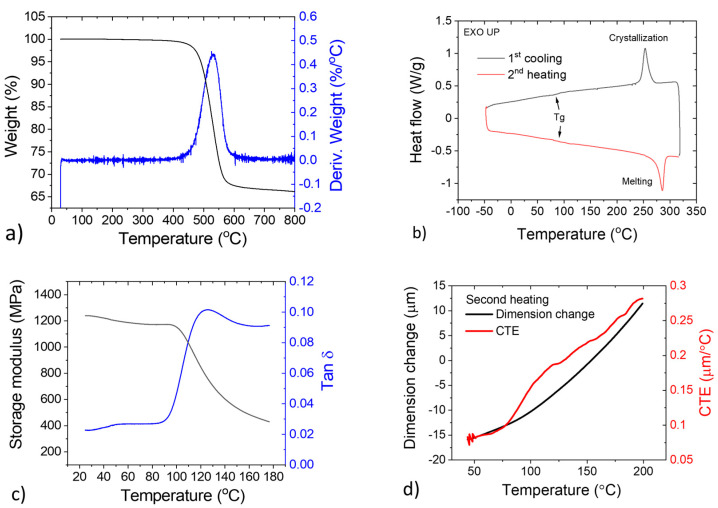
Thermal analysis of the polymer heat exchanger. (**a**) TGA curve showing a maximum decomposition rate around 531 °C; (**b**) DSC curve showing a T_g_ of 87.6 °C and a T_m_ of 285.6 °C; (**c**) DMA oscillation showing the evolution of storage modulus and tan δ with temperature; and (**d**) TMA curve showing excellent dimensional stability of the polymer composite.

The results of the TGA analysis are presented in Figure 4a. The graph shows the thermal decomposition behavior of the polymer composite. A maximum decomposition rate of 0.44% per °C was observed at 531 °C. The sample also shows a high char yield due to the graphitic fillers and the aromatic structure of PPS [36,37]. For the DSC results, a glass transition temperature (T_g_) of 87.6 °C and melting temperature (T_m_) of 285.6 °C were observed, as indicated by the step change and the sharp peaks, respectively, of the heat flow curves in both heating and cooling in Figure 4b. It should be noted that the step change in the DSC curve reflecting the glass transition of the material was overshadowed by the melting peak. Figure 4c shows the change in the storage modulus and tan δ of the material as a function of temperature in an oscillatory DMA experiment. A sharp decrease in the storage modulus is observed, corresponding to the glass transition of the polymer matrix. A T_g_ of 125.3 °C was determined from the peak of the tan δ curve. The values of T_g_ measured by DSC and DMA are different, which is not unexpected because the underlying property being monitored was not the same. The T_g_ measurement in DSC involves monitoring a thermodynamic property (heat capacity), whereas the T_g_ in DMA is obtained from a viscoelastic property (tan δ). Figure 4d shows thermal expansion behavior of the polymer composite characterized using TMA. The CTE exhibits a noticeable change in the slope when the temperature exceeded ~80 °C, indicating the glass transition. Furthermore, the composite exhibited excellent dimensional stability with CTE values less than 0.1 μm/°C below the glass transition. The thermal analysis results indicate that the polymer matrix has a semicrystalline structure with high thermal and dimensional stability suitable for heat exchanger applications. 

### 3.3. Surface Morphology

**Figure 5:**
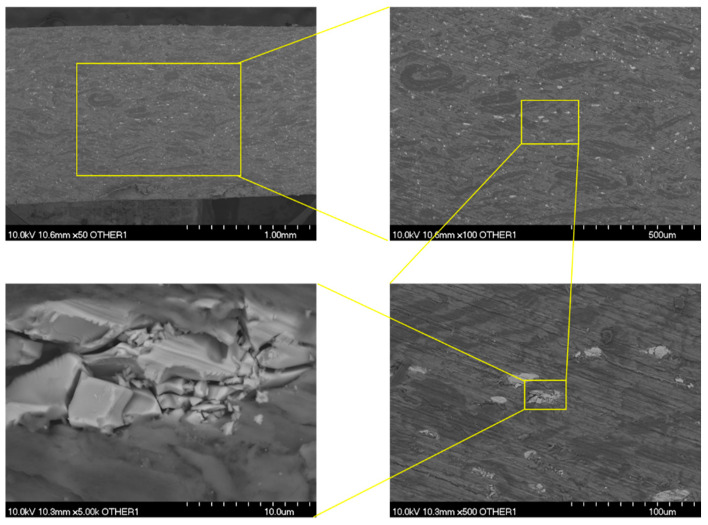
SEM images showing a multi-phase structure of the as-received sample.

**Figure 6:**
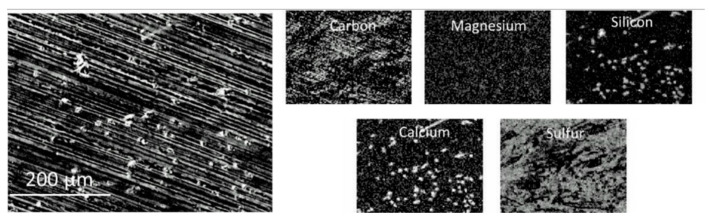
SEM-EDS results that show the presence of elements such as carbon, magnesium, silicon, calcium, and sulfur in the as-received sample.

The results of the SEM characterization of the as-received sample are presented in Figure 5. A three-phase structure can be observed. This structure consists of a polymer matrix, a graphitic filler (black strips), and an inorganic filler (white dots). Results of the EDS analysis (see Figure 6) reveal that besides carbon and sulfur, magnesium, silicon, calcium were present on the surface. The graphitic and inorganic fillers are thought to enhance the thermal conductivity and mechanical properties of the polymer, respectively [38,39,40,41]. For instance, CaCO_3_ has been found to improve the tensile, impact resistance, and flexural strength of certain polymers [40,41]. 

### 3.4. Creep Test and Modeling

**Figure 7:**
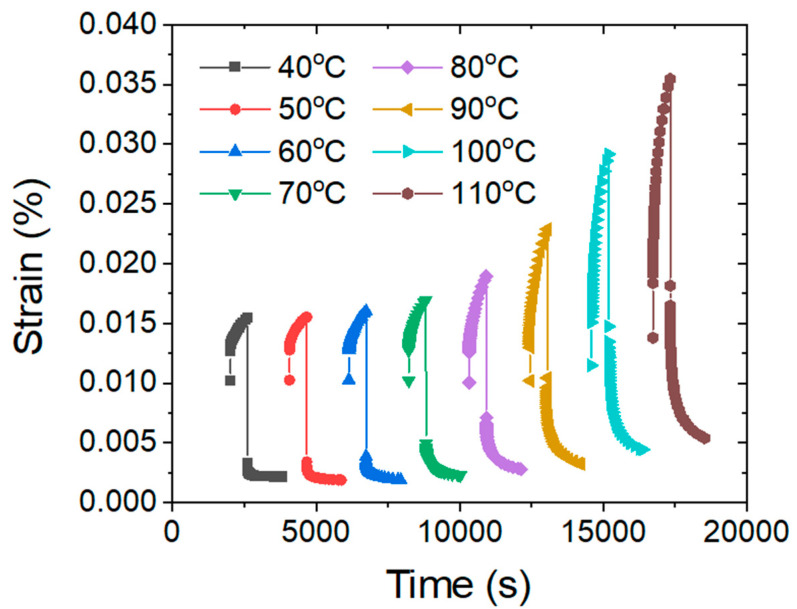
Creep and creep recovery curves at test temperatures ranging from 40 to 110 °C.

Figure 7 displays the results for the creep and creep recovery tests performed at isotherms from 40 to 110 °C in intervals of 10 °C. The creep behavior of the polymer composite exhibits a strong temperature dependence, which is caused by the influence of the glass transition. At low temperatures (40–70 °C), the polymer is in a glassy state and the movement of the polymer chains is greatly restricted, implying that there was creep resistance. Therefore, the material exhibits a low and almost constant creep strain value of 0.015%. The creep strain value gradually increases from 0.02% at 80 °C to 0.35% at 110 °C, indicating the transition of the material from a glassy state to a rubbery state. At 80 °C, branches and shorter polymer chains are thermally activated and start to show flow behavior; while at 110 °C, longer polymer chains become soft and unentangled, allowing larger deformations. This gradual increase in creep strain from 80 to 110 °C agrees with the broad glass transition observed in the DSC experiment.

**Figure 8:**
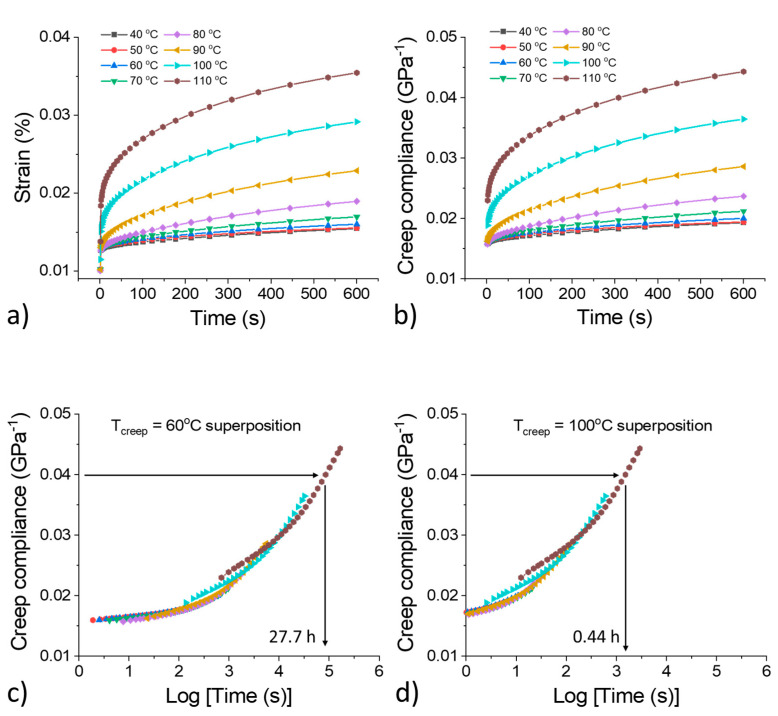
Creep behavior of the polymer composite. (**a**,**b**) Time-dependent creep strain and creep compliance at different temperature intervals. (**c**,**d**) Master curves generated from manually shifted creep compliance data based on a time–temperature superposition principle, which predicts the long-term performance of the material.

The time-dependent creep strain and creep compliance values at different temperature intervals are shown in Figure 8a,b. The data monotonically increase with respect to the test temperature. At temperatures below T_g_, the movement of the polymer chains is greatly restricted and thus the material exhibits limited creep strain behavior. Assuming that carbon nanotubes are present in the matrix, the resistance of the polymer to creep at relatively low temperatures (40–70 °C) may be due to certain alignments of the polymer chains and carbon nanotubes that effectively restrict the polymer chains from stretching, rotation, refolding, disentanglement as well as sliding under loading [42]. At temperatures above T_g_, on the other hand, the material is thermally activated, allowing for larger deformation. Figure 8c,d presents the master curves that were generated from the manually shifted creep compliance data using 60 °C and 100 °C as the reference temperature, respectively. For example, when constructing the master curve for 60 °C, all the creep compliance data (Figure 8a) collected at different temperatures (40 °C to 110 °C) were first replotted on a log scale. Then, the data collected at temperatures lower than 60 °C were manually shifted to the left and the data collected at temperatures higher than 60 °C were manually shifted to the right to form a smooth curve, which represents the long-term performance of the materials when operating at 60 °C. For a given creep compliance value, 0.04 GPa^−1^ for example during use at 60 °C, the polymer can stay functional for 27.7 h, whereas during use at 100 °C the service time drops to 0.44 h. Such a drop in the service time indicates that at 100 °C, the polymer is highly susceptible to creep, and therefore should not be used at this temperature.

**Figure 9:**
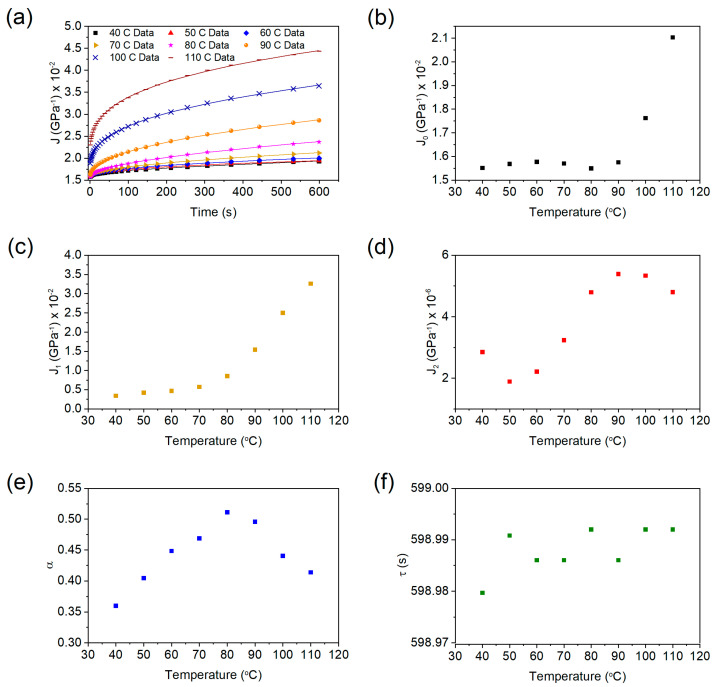
The fitted values for (**a**) *J*(*t*), (**b**) *J*_0_, (**c**) *J*_1_, (**d**) *J*_2_, (**e**) *α*, and (**f**) *τ* for test temperatures ranging from 40 to 110 °C.

Figure 9a features the graph for the creep compliance data, *J*(*t*), and the corresponding fitted curves. As can be seen, the fitted curves display an excellent fit to the data. Figure 9b–f displays the fitting parameters (from Equation (1)) *J*_0_, *J*_1_, *J*_2_, *α*, and *τ* as a function of the testing temperature. The creep compliance coefficients *J*_0_ and *J*_1_ increased significantly for temperatures greater than 80 °C. The increase in the compliance values *J*_0_ and *J*_1_ with temperature are likely due to the increasing flexibility of polymer chains [43] as the temperature approaches the T_g_ (around 125.3 °C) of the polymer, as confirmed by the DMA result. Furthermore, this increase in the flexibility of the chains is accompanied by a decrease in the amount of stored elastic energy as well as a decrease in the storage modulus values [44], as found in Figure 4c. Also, these moduli followed a similar increasing trend as tan δ. The value *J*_2_ also exhibited an increase (nonmonotonic) over the prescribed temperature range. This increase suggests that the polymer chains exhibited a greater amount of permanent deformation as the temperature increased towards T_g_. 

The Kohlrausch coefficient, on the other hand, initially increased until it attained a maximum value at 80 °C, and then subsequently decreased in value. The Kohlrausch coefficient accounts for the distribution of relaxation times and varies between 0 (representing a single relaxation time) and 1 (a wider distribution of relaxation times) [45]. The observation that the Kohlrausch coefficient peaked at 80 °C can be explained by the glass transition of the polymer. As the material passes through the glass transition, the polymer chains soften, resulting in a wider distribution of relaxation times. Before or after the glass transition, however, the polymer chains are completely frozen or relaxed, resulting in a narrower distribution of relaxation times. The retardation time did not show noticeable change within the investigated temperature range.

### 3.5. Nanoindentation Behavior

**Figure 10:**
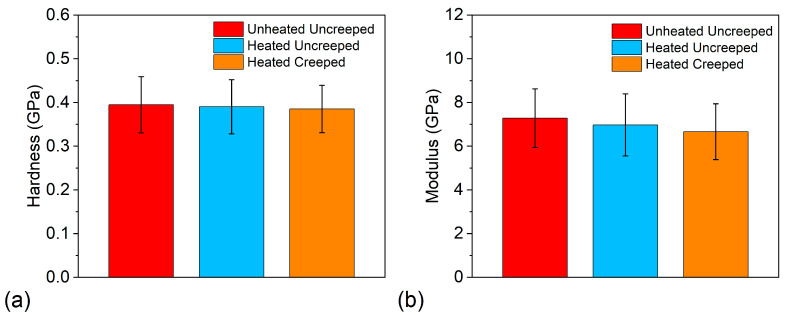
The nanoindentation (**a**) hardness and (**b**) Young’s modulus for the unheated and uncrept, heated and uncrept, and heated and creeped samples indented to a maximum displacement of ~1000 nm.

Figure 10 displays the nanoindentation hardness and Young’s modulus results for the unheated-uncrept, heated-uncrept, and heated-creeped samples that were indented in the displacement control mode to a maximum displacement of ~1000 nm. As can be seen, the average hardness and Young’s modulus values range from 0.39 to 0.40 GPa and 6.66 to 7.28 GPa, respectively (see Table 2). Furthermore, the values are within one standard deviation from one another, indicating that the creep deformation and the heating did not significantly change the nanoindentation properties of the polymer. This finding also indicates that there were no significant changes to the crystallinity of the PPS matrix and filler content. Importantly, these results suggest that the polymer can retain its mechanical and microstructural properties when exposed to the stress and thermal conditions typical of a heat exchanger. 

## 4. Conclusions

In this investigation, the structural, thermal, and mechanical properties of a graphite containing a polymer composite were examined using various experimental techniques. FTIR and XRD characterization revealed the presence of graphitic fillers in a PPS-based polymer matrix. Moreover, the DSC results showed that the T_g_ of the polymer was 87.6 °C, indicating that the material is potentially suitable for heat exchanger applications. Creep tests were performed at different isotherms to enable the use of TTSP to predict the long-term performance of the material, which was found to be highly temperature-dependent. Furthermore, the fitting of the time-dependent creep compliance data correlated well with the dynamic mechanical analysis, indicating that the composite can retain its mechanical properties when used below its glass transition temperature. Nanoindentation tests suggested that the mechanical behavior of the composite was not significantly affected by temperature or plastic deformation. Based on the results of this study, the composite could be used for heat exchanger applications where low cost and high design flexibility are required.

Notice: This manuscript has been authored by UT-Battelle, LLC under Contract No. DE-AC05-00OR22725 with the U.S. Department of Energy. The United States Government retains and the publisher, by accepting the article for publication, acknowledges that the United States Government retains a non-exclusive, paid-up, irrevocable, world-wide license to publish or reproduce the published form of this manuscript, or allow others to do so, for United States Government purposes. The Department of Energy will provide public access to these results of federally sponsored research in accordance with the DOE Public Access Plan (http://energy.gov/downloads/doe-public-access-plan, accessed on 13 June 2021).

## Figures and Tables

**Table 1:** XRD and FTIR peak assignment results for the characterized polymer composite.

XRD		
2 Theta Angle (°)	d-Spacing (Å)	Diffraction Plane
18.9	4.69	(110) of PPS
20.5	4.33	(102), (200), (111) of PPS
26.6	3.35	(002) of graphite
36.3	2.47	(004), (302), (311), (022), (104), (213) of PPS
54.7	1.68	(004) of graphite
**FTIR**		
**Wavenumber (cm**^**−1**^)	**IR Fingerprint**	
3050–3150	Aromatic C-H stretching	
1650–1750	Aromatic C-H bending	
1380, 1470, 1573	Aromatic C=C stretching	
1060,1091	C-S stretching	
1009, 804	Aromatic C–H bending	

**Table 2:** The hardness and Young’s moduli of the unheated and uncrept, heated and uncrept, and heated and creeped specimens at a maximum displacement of ~1000 nm.

Sample	Hardness (GPa)	Young’s Modulus (GPa)
Unheated and uncrept	0.40 ± 0.06	7.28 ± 1.34
Heated and uncrept	0.39 ± 0.06	6.97 ± 1.42
Heated and crept	0.39 ± 0.05	6.66 ± 1.28
